# Supplementary material for: The Contribution of Pharmacists and Pharmacy Technicians to Person-Centred Care within a Medicine’s Optimisation in Care Homes Service: A Qualitative Evaluation
Source: Pharmacy (Basel). 2021 Feb 10;9(1):34. doi: 10.3390/pharmacy9010034 (PMC7931069; doi:10.3390/pharmacy9010034)
Supplement: Supplementary file 1 [file pharmacy-09-00034-s001.pdf]

Figure S1: How the MOCH intervention has enabled person-centred care

	Enablers	Barriers
**Authentic Consciousness**	MOCH HCP considering overall state of resident	Previous rapid medication reviews poorly communicated
	Understanding resident medication routines	
A consideration of the person's life as a whole in order to help sustain meaning in life.	MOCH HCP understanding effects of medication on resident's carers/family	
	Tailoring MOCH response for specific care home	
	Observing care home procedures before intervening	
	MOCH HCP understanding of resident's health values	
	Undertaking full review (medication and lifestyle) with all residents	
	Awareness of other agencies/clinical teams in care home	
**Informed flexibility**	Educating care home staff on lifestyle contributors to health	Residents not aware of purpose of medicines
	Education to care home staff concerning medications (side effects, expiry dates)	Care home variation of knowledge re understanding/dispensing of certain medications
The facilitation of decision making through information sharing and the integration of new information into established perspectives and care practices.	Educating care home staff on medication processing IT systems	
	Improving lifestyle' education to residents	Resident unaware of different medications administered to them
	Signposting to clinical/non clinical services if MOCH cannot support	
	Educating GPs on polypharmacy/deprescribing	
	Educating GP surgery in care home staff competencies (e.g. taking BP)	
	Sharing intervention experiences with MOCH team	
	Educate family on effects of medication and lifestyle choices on resident health	
	Information of resident medication processes given to new care home when moved	
**Mutuality**	Involving other healthcare teams in decision making	Weak relationship between resident's family and care home staff
	Good working relationship with GP	Primary care unreceptive to MOCH team, refusing access to SystmONE
The recognition of the others' values as being of equal importance in decision making.	Resident open to discuss meds with MOCH team	Varied receptiveness of care home managers to MOCH initiative, restricting access to MOCH team.
	Liaising with other non-clinical staff in care home (e.g. chefs)	
	Awareness of care home view on MOCH project	
	Positive working relationship with care home	
	Non-judgemental approach to current care home processes	
	Gaining family input on resident care where possible	
	Education to CH staff of importance of resident home environment	
	MOCH HCP recognising care home staff needs and values	
**Transparency**	Implementing or updating online medication system	Unclear resident medication notes
	Clear documentation re non-pharmacological interventions	Medication processing not up to date/unsynced between staff
Making explicit the intentions and motivations for action and the boundaries within which care decisions are set.	Clearly communicating MOCH aims to all parties involved	MOCH teams operating locally, no person to escalate barriers or decision making
	Using all assessments to formulate centralised plan of care	Little care home communication with resident
	Follow up with resident/family important to establish	Large numbers of healthcare workers involved in resident decision making
	Constant line of communication with family/carers on drug side effects/changes	MOCH team member absence
	Establishing introduction with care home and primary care early on	MOCH team confusion on which CCG to liaise with
	Unified communication between all parties involved in resident care	Time taken for new service to establishing communication between clinical teams/primary care networks
	Frequent visits to see and speak to residents	
	MOCH team working towards same goals internally	Previous medication administration system not been successful
	Open door policy for care home staff and residents to discuss MOCH	
	Communication on what care home is doing well	
**Negotiation**	Agreement from all parties involved in resident care is important	Patient unresponsive to MOCH team member (not compos mentis) - unable to participate
A culture of care that values the views of the patient as a legitimate basis for decision making while recognizing that being the final arbiter of decisions is of secondary importance.	Resident-led communication and changes	
	Addressing family concerns	
	Addressing resident medication concerns/issues with health	
	Family and care home involvement if resident is not compos mentis	
**Sympathetic Presence**	Reviewing residents in sensitive manner	Resident previously overlooked by HCP
An engagement that recognizes the uniqueness and value of the individual by appropriately responding to cues that maximize coping resources through the recognition of important agendas in daily life.	Tailoring discussion to resident's needs	
	Giving residents autonomy over own health choices	
	MOCH team taking time to help resident with everyday needs	
	MOCH team member advocating for resident's wellbeing	
	Taking time to get to know residents	
	Working to tailor intervention to resident	
**Patient/resident's values history**	Residents feeling they were not 'forgotten'	Apprehension around medication changes
		Lack of resident's confidence to ask for changes
**Context of care environment**	Supportive and present family	MOCH HCP unclear about the imperative to reduce cost through deprescribing
	Care home staff's education on medications	Care home culture around resident autonomy
	Good working relationship with GP practice	High turnover in care home staff
	Type of care home (residential, nursing)	Covering MOCH over large geographical areas
	MOCH team working within a primary care setting (e.g. GP surgery)	Multiple care groups to liaise with
	Care home having good prior working procedures	Care homes being a 'forgotten entity'
	Time to implement initiative	Continuing change in the organisation of MOCH.
	CCG targeting suitable care homes	
**Practitioner or carer values history, knowledge and experience**	Experience of assessing residents/patients medication and state	Different clinical system challenge for nurses (pre-intervention)
	Experience of assessing lifestyle factors in addition to medication	Challenging behaviour of resident
	Previous experience of working in care homes or community setting	
	Experienced consultation skills	
	Observation of care home processes	
	Care of patient always at forefront of practice	
	Enjoyment of patient/resident-facing role	
**Resident outcomes**	Improved physical state	
	Improved mental state/mood	
	Higher frequency of resident socialising	
	More engagement in future medication recommendations	
	Better pain management	
	Lower resident fall risk	
	Higher resident independence	

	Better diet and hydration
	Improved confidence in taking medications
	Reduced embarrassment from medication side effects
**Carers/Family outcomes**	Care staff can spend more time with residents in more need
	Care staff confidence in ordering medications
	Carers aware of lifestyle changes affecting residents
	Care home staff understanding of patient involvement in medication review
	Improved staff awareness around medication wastage
	Better established relationships between carer and resident
	Family awareness of medications
	Reduced family distress
**Organisational outcomes**	Reduction in care home medication mistakes
	Less resources and staff needed to care for resident
	Improved information flow from care home to GP surgery
	Reduction in medication errors
	Improved education on medications
	Improved medication storage
	Less GP or primary care visits to care home
	Improvement in care home safety
	Reducing hospital admissions
**MOCH team member outcomes**	Growth in experience in community settings/care home settings
	Adapting to different work environments
	Further training opportunities (e.g. prescribing)
	Improved clinical experience
	Improved confidence in medicine reviews
	Improved team communication skills and cohesion
	Appreciation of primary care roles
	Confidence to grow in career
